# A rapid review examining purchasing changes resulting from fiscal measures targeted at high sugar foods and sugar-sweetened drinks

**DOI:** 10.1038/s41387-017-0001-1

**Published:** 2017-12-15

**Authors:** Katharine E. Roberts, Louisa J. Ells, Victoria J. McGowan, Theodora Machaira, Victoria C. Targett, Rachel E. Allen, Alison E. Tedstone

**Affiliations:** 1 Health Improvement Directorate, Public Health England, Skipton House, 80 London Road, London, SE1 6LH UK; 2 Health and Social Care Institute, Teesside University, Middlesbrough, TS1 3BA UK; 3 Centre for Health Inequalities Research, Department of Geography, Durham University, Durham, DH1 3LE UK

## Abstract

To aim of the review was to examine the most recent (2010 onwards) research evidence on the health and behavioural impacts, in adults and children, of fiscal strategies that target high sugar foods and sugar-sweetened drinks (SSDs). A pragmatic rapid review was undertaken using a systematic search strategy. The review was part of a programme of work to support policy development in relation to high sugar food and SSDs. A total of 11 primary research publications were included, describing evidence from France (*n* = 1), the Netherlands (*n* = 3), and the United States of America (*n* = 7), assessed through a variety of study designs, with the majority in adult populations (*n* = 10). The evidence reviewed focused on consumer behaviour outcomes and suggested that fiscal strategies can influence purchases of high sugar products. Although the majority of studies (*n* = 10), including three field studies, demonstrated that an increase in the price of high sugar foods and SSDs resulted in a decrease in purchases, eight studies were conducted in a laboratory or virtual setting which may not reflect real-life situations.

Findings from this review support evidence from the broader literature that suggests that fiscal measures can be effective in influencing the purchasing of high sugar foods and SSDs.

## Introduction

The UK population consumes more sugar than is recommended^1^ and sugar consumption increases the risk of consuming too many calories which contributes to weight gain and obesity^2^. As an important determinant of food choice, price is one focus for interventions aimed at changing population level dietary consumption^3^. Price based initiatives such as taxes, subsidies and other economic initiatives are employed in some countries, either to discourage the consumption of unhealthy nutrients such as salt, sugar and saturated fat or encourage the consumption of healthy foods such as fruit and vegetables.

Taxes can be applied as a sales tax (applied at point of purchase as a proportion of the value of the good) or an excise tax (typically per unit and applied on the sale or production for sale of the good), on a specific nutrient, a combination of nutrients or on a category of food or drink such as sugar sweetened drinks (SSDs)^4^. A tax on SSDs in particular has been of interest because of their association with obesity, diabetes^5^ and dental caries^6^. SSDs contribute a significant proportion of sugar consumed in the UK particularly by children and young adults (up to 30% of energy from sugar in teenagers)^7^. As a contributor to diet related ill health, frequently with little nutrient value other than calories from sugar and with readily available substitutions in the form of either diet drinks or water, they are currently a target for taxation in many countries^8^.

Interventions aimed at changing population level, dietary behaviours are complex and comprise multiple interacting components. Large scale randomised controlled trials are regarded as the gold standard for evaluating the effectiveness of complex interventions^9^. However, these are not always feasible due to time and financial constraints. In the peer-reviewed literature, the evidence of effectiveness of health-related taxes focused on food and drink is in the form of natural experiments, experiments in controlled environments and modelling studies^10^. These study designs have different strengths and limitations, particularly in relation to their internal or external validity. This review focuses only on experimental and observational studies.

Evidence from modelling studies have been examined elsewhere in several high quality systematic reviews^11–14^. These studies are considerably heterogeneous with regards to the levels and nature of taxation, the outcomes that they investigate, the data sources employed, the analytical approaches and the modelling assumptions applied^15^.

A few studies undertaken in the US where there are existing excise taxes on SSDs employ empirical data, for example, sales and excise tax (price) data, and merge them with cross-sectional or longitudinal data relating to consumption or health outcomes, using temporal or geographical identifiers, to identify associations between the data^16, 17^. Several studies have then extrapolated from these effects on sales to purchases, to estimate the effect on health outcomes such as body mass index (BMI) and have predicted small impacts^18–20^. However, many of these studies are set in States where the levied tax levels are low (<10%). In addition, studies of this type are subject to a range of confounders and biases such as variance between population level obesity prevalence or socio-demographic distribution which can weaken the case for causality if adjustments in the analyses methods are not made^21^.

Many studies investigating the impact of taxes have used econometric modelling techniques to estimate price and other demand elasticities and predict or simulate the effects of various tax scenarios on consumption or sales using existing previously reported data^15, 22–25^. Reviews of the evidence from these types of studies suggest that a tax of 10 to 20% would be necessary to have a significant impact on purchases, consumption and ultimately population health^10, 11, 26, 27^. With reference to SSDs specifically, reviewers have concluded that reductions in purchasing are proportionate to increases in price at the consumer level^12, 14, 26^. A systematic review of modelling studies identified 16 studies with taxes on SSDs ranging from an increase of 5 to 30%. The results from all included studies showed a reduction in consumption of SSDs that ranged from 5 to 48% with the overall pattern demonstrating that the reductions were proportional to the tax applied^12^.

One systematic review by Epstein, examined only experimental research on the relation between food price changes and food-purchasing patterns. The review included 24 studies examining changing levels of purchasing of categories of foods defined by a range of criteria broadly related to health impact, including the proportion of sugar in the food and drink products (e.g., healthy/less healthy, high/low energy density, high/low calorie for nutrient, regular/diet soft drinks) from January 1980 until March 2011 (therefore many studies were outside the dates for inclusion criteria for this review)^28^. These studies were of varying quality. However, it is frequently not feasible to conduct experimental economics research using study designs that follow a gold standard biomedical research model such as for randomised controlled trials (RCTs). The review concluded that the experimental evidence suggests that price changes do modify purchases of targeted foods. However, the overall impact on the nutritional quality of dietary intake is unclear due to the largely unknown potential effects of consumers substituting purchases of foods/drinks of a particular nutrient composition, for example, high sugar with foods/drinks of a different nutrient composition, for example, high fat. More complex research is therefore needed in this area^28^.

**Fig. 1:**
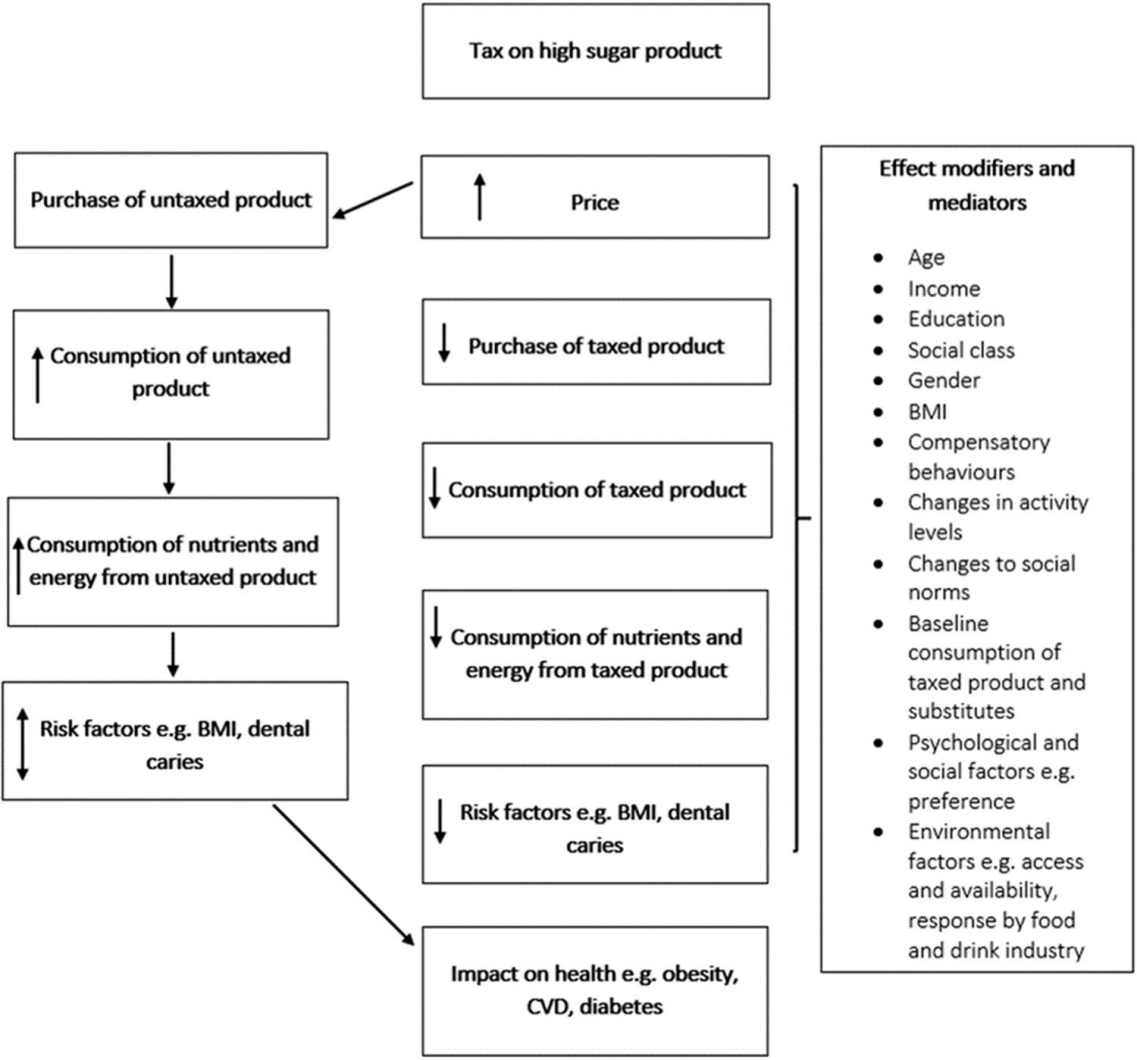
**Hypothesised logic model for the possible effect of a fiscal strategy on high sugar products. Source: Adapted from Mytton et al., 2014** ^21^

The complex nature of diet related behaviour and its association with health outcomes such as obesity should be carefully considered in terms of how a tax on high sugar foods and drinks is implemented. Food consumption and its association with health outcomes is non-linear and is influenced by a diverse set of determinants that operate and interact at an individual, community and population level^21, 29^. Figure 1 illustrates the logical framework for how product price may affect behaviour and, in turn, health outcomes. It also shows the numerous mediators and modifiers that may influence behaviour change and potentially lessen the impact of a tax. There is evidence from both experimental and modelling studies that adverse substitution or compensatory effects from taxation of foods and drinks can occur. For example, taxing one food or nutrient may be offset by substitution with other nutrients that also have negative or no positive consequences for dietary quality overall^27^. However, these effects may be mitigated when healthier alternatives to the taxed food or drink are available, for example sugar-free alternatives to SSDs.

### Aim

The aim of this review was to examine the most recent (2010 onwards) research evidence on the health and behavioural impacts of fiscal measures targeted at high sugar foods and SSDs in both adult and children populations.

## Methods

### Inclusion criteria

Studies from 2010 onwards were selected to provide an overview of the most current research evidence to fit the resource and policy requirements outlined in the research brief (Appendix 1). Included studies had to meet the following:

- Population: studies involving populations of any age (children were defined as <18 years of age; adults 18 years and over), from the Organisation for Economic Co-operation and Development (OECD) countries (to enhance the applicability of findings to the UK).
- Outcomes: consumption patterns, purchasing patterns, and intake, preferences, excess weight, weight gain, dental health, diabetes, cardiovascular disease risk, attitudes, energy.
- Intervention: Any experimental or observational study that demonstrated a health or behavioural impact of a fiscal strategy on high sugar foods and/or SSDs. (For the purposes of this review the definition of ‘high sugar foods’ is broad to reflect the diversity of the outcome measures in the literature, the nature of sugar consumption in the diets of free living individuals and the aims of the review. Studies are included that examine the impact of fiscal measures on differing categories of foods, grouped by criteria that include high sugar content (e.g., high/low calorie, high/low energy density, healthy/less healthy). Studies are included where food categories were based on established 'cut-offs' or where foods typically high in sugar (e.g., confectionery) were categorised and examined against foods typically low in sugar (e.g., vegetables). Relevant studies examining 'sugar sweetened drinks' or 'regular soft drinks' as an outcome were included).
- Exclusion criteria:
- Commentaries, systematic reviews, non-systematic reviews, qualitative studies, modelling studies, or discussion pieces.
- Research that focused on nutrition labelling, health promotion or the promotion of healthy food/drink.
- Non-English language papers.
- Studies published outside of stipulated publication dates.
- Studies from non OECD countries.
- Studies with no outcome data relating to consumption or purchasing of high sugar foods or drinks.
- Studies focused on alcohol.

#### Search strategy

A list of key search terms (see Appendix 2) was developed by the project team in consultation with the steering group. The steering group consisted of 16 members including the Public Health England and University of Teesside project team, representatives from the Department of Health, HM Treasury, key non-governmental organisations and academic experts in the field. The purpose of the steering group was to define, review and agree the research questions, search strategy, methods and final outputs. Each electronic database (CINAHL, Cochrane library, Embase, Health Business Elite, HMIC, LILACS, Medline, and PsycInfo) was systematically searched using a combination of these terms, tailored to optimise sensitivity, specificity, and the syntax and functionality of each database. The final search strings were created and run on 30 October 2014 by an information scientist. An example search string is shown in Appendix 2. The database search results were also supplemented by hand searches, and references provided by the steering group, stakeholder interviewees and ongoing study author contacts.

In addition to the peer reviewed literature, key government and organisation websites as well as general sites such as Google, Bing and the social media sites Facebook and Twitter were searched for grey literature using the broad search term: 'sugar and food and drink'. A full list of the 'grey literature' (non peer-reviewed) searches are shown in the Public Health England (PHE) evidence review^30^.

#### Screening and data extraction

All titles and abstracts were screened by one reviewer. The resulting shortlist was reviewed by the research team, to finalise the list of references that potentially met the inclusion criteria. Full text versions of these papers were extracted and assessed by one reviewer, and a second reviewer was consulted where any ambiguity existed. Any conference proceedings or study protocols were categorised as ongoing studies, and where contact details were available, authors were contacted for further information (details are provided in the PHE evidence review^30^). The final list of included papers was reviewed by the project steering group (membership details included in the PHE evidence review^30^) to ensure the research brief was fulfilled and all key publications had been captured.

The literature was originally screened to extract those studies which specifically examined ‘high sugar’ foods and drinks. However, a steering group decision was made to also include those studies meeting the inclusion criteria that reported on a range of foods or categories of foods providing they included, or referred to, a high sugar component. This better reflected the diversity of outcomes in the literature and the consumption of sugar in a free living environment as a component of a food or meal.

Data extraction and quality appraisals (see Appendix 3 and 4) were carried out for each included study by two reviewers, using the Joanna Briggs Institute appraisal tools as guides, which were adapted to meet the pragmatic requirements of the review^31^. A third reviewer was consulted if any queries arose. Based on how well each study met the quality appraisal criteria, a subjective overall quality category (low, moderate or high) was assigned. Due to the vast heterogeneity of the included studies, meta-analyses were not possible, therefore a narrative synthesis is provided. Evidence was appraised by examining the context of the study quality and consistency of findings. Key findings were contextualised within the study design, quality assessment, and objectivity of the outcome measure.

## Results

**Fig. 2:**
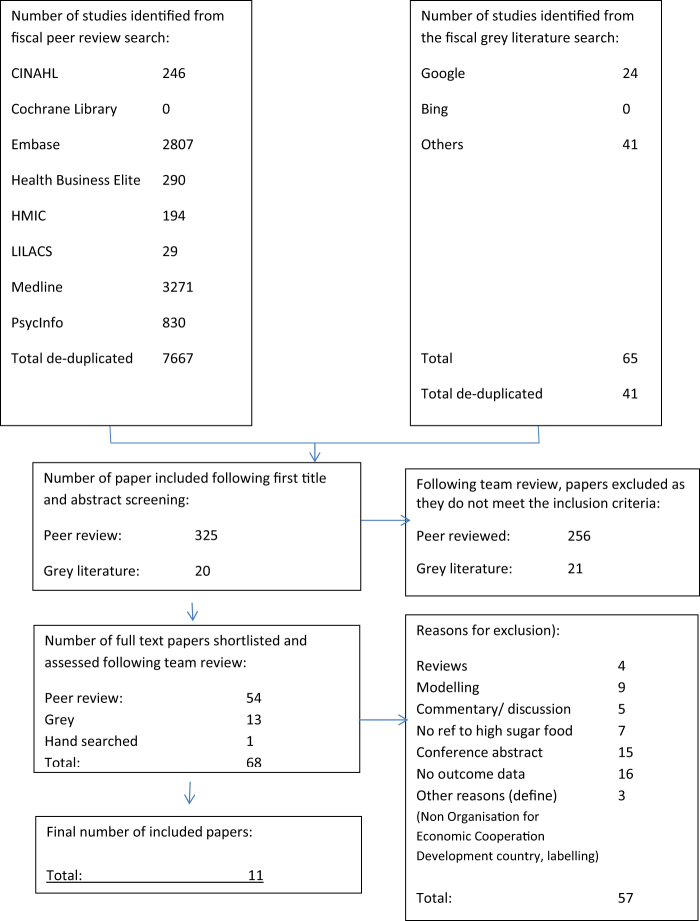
**Fiscal literature flow diagram**

A flow diagram summarising the search and sifting results is shown in Fig. 2. Of the 11 studies included in this review, 10 were conducted in adult populations and one was conducted in children. Data summary tables are presented in Appendix 4. The 11 primary studies were conducted in France (*n* = 1), the Netherlands (*n* = 3) and the US (*n* = 7) and were largely experimental in either a laboratory (*n* = 4), virtual setting (*n* = 4) or controlled field experiments in supermarkets (*n* = 2) or a cafeteria (*n* = 1). The majority of studies were small in scale with seven studies having sample sizes of *n* < 200. Study quality was generally moderate with many of the studies lacking details about blinding, allocation concealment and withdrawals so they failed to gain higher scores on the quality assessment model applied (see Appendix 3 and 4). Declarations of funding source for each study show, where declared (in 6/11 studies), that funding was derived from research councils or foundation trusts. No explicit commercial funding was declared. The studies represent data from experimental and observational studies with a variety of study designs, locations, populations, outcome data and data collection methods and a wide range of product and food outcomes. Very few studies provided nutritional analysis of the target products assessed. The vast majority of included studies reported outcomes related to sales/purchases. These behaviours, therefore, provide the focus of the narrative comparisons presented in this review, as it was not possible to conduct any meta-analyses given the heterogeneity between studies.

### Laboratory/virtual experiments

There were eight studies conducted in a laboratory (*n* = 5, 1 of which was controlled)^32–35^ or virtual, that is, web-based shopping setting (*n* = 3, 2 RCTs, 1 controlled no randomisation)^36–39^. Seven of these studies were carried out in adult only populations^33, 34, 36–39^ and one study was carried out in children aged 12 to 14 years^35^. Seven studies demonstrated that an increase in prices of SSDs or groups of 'unhealthy' or 'energy dense' foods and products (including those with ‘high sugar’ content) resulted in a decrease in purchases^32–37, 39^ with one study showing no effect^38^.

An RCT by Waterlander^37^, was conducted using a virtual supermarket in the Netherlands and focused on purchases of SSDs. Following an increase in Value Added Tax on SSDs from 6 to 19% (a mean change of 12.4%) results showed a statistically significant decrease in the purchase of SSDs of 0.9 litre per household per week in the intervention group vs. control^37^. This study was relatively small (*n* = 102) but of high quality. A French study by Darmon^33^ (*n* = 33) employed a combined 'subsidy' of 30% on 'healthy' foods and a 30% 'tax' on 'unhealthy' foods (defined by measures of nutritional quality: calories per 100 g, percentage of free sugars, percentage of recommended intakes for several key nutrients or Mean Adequacy Ratio (MAR)). There were no ‘tax only’ condition outcome data and the effect on sugar intake was not reported, however a reduction in purchases of ‘unhealthy’ foods due to was reported^33^. This was the only study to examine the different impacts of the intervention in low and medium income groups. While the intervention resulted in an improved nutritional quality of foods purchased overall in both income groups, the extent of the improvement was greater in the middle income group than the low income group suggesting that price manipulations would not necessarily tackle income driven inequalities in nutritional quality of food purchases.

A virtual RCT by Waterlander^38^ (*n* = 117) examined the purchasing effects of three price levels on 'unhealthy' foods (increases of: 5%, 10%, 25%) and on 'healthy' foods (decreases of: 0, 25%, 50%). A factorial study design was used to examine the effect of different combinations of increase and decrease. Regression analysis was undertaken to assess the overall effect of the 'tax only' condition and no effect on purchases of 'unhealthy' foods was reported. The results indicate the complex nature of compensatory behaviour, as although those with the highest discount on healthy foods purchased significantly more healthy foods than the other groups, they also purchased more calories overall.

A US descriptive study by Epstein^34^ also examined the effect of increasing the price of 'unhealthy' foods and lowering the price of 'healthy' foods but each condition was tested separately in a group of mothers (*n* = 42). A price increase of 10% on ‘unhealthy’ foods resulted in a 14.4% reduction in purchases of these foods and a 6.5% reduction in total calories purchased. Interestingly, subsidies of ‘healthy’ foods did not result in a decrease in total calories purchased overall as mothers spent the saving from healthy food on more unhealthy food, again providing some insight into the potentially unexpected substitution effects of price manipulations.

In the US, a cluster-randomised, controlled laboratory study by Giesen (*n* = 178)^36^ examined the effect on purchasing of calorie labelling alongside increased prices of high calorie foods and drinks and employed three levels of 'taxation' (none; 25 and 50%) but also added other factorial layers by either providing the participants with $10 or $20 ('high' or 'low' budget) and adding calorie information or not. A taxation of 25 or 50% on high calorie foods had a significant main effect in reducing calories purchased (estimate: −0.780, *p* < 0.001).

A controlled virtual study in the Netherlands (*n* = 306) by Nederkoorn^39^, found that a 'tax' on energy dense foods (50% on products with a caloric value of >300 kcal/100 g) resulted in 16% fewer energy dense foods and 8% fewer total calories being purchased. These results were not influenced by BMI. Another experimental within-subject study, by Temple in New York (*n* = <100) employed a 25% tax on foods which were higher in calories, sugar (>25% calories/serving) and fat (>5 g fat/serving). These foods were labelled as 'red' within a 'traffic lights' categorisation method. This reported that there was a significant main effect of taxation in relation to reducing purchases of ‘red’ foods (no data given) with taxation reducing the purchasing of ‘red’ foods. This reduction was observed in obese participants but not in non-obese participants^32^.

The only study conducted with children (*n* = 89), by Salvy^35^, was descriptive and examined the effect of 'unhealthy' and 'healthy' snack food price manipulation on the purchases of a sample of children aged 12 to 14 years. 'Healthy' and 'unhealthy' were defined by low/high scores on a calorie-for-nutrient index which calculates the number of calories needed to gain 1% of the recommended daily values of 13 key nutrients. Purchases of ‘unhealthy’ snacks decreased and purchases of 'healthy' snacks increased when the price of unhealthy snacks were taxed^35^.

### Supermarket/cafeteria/restaurant experiments

Two studies, a randomised controlled field experiment^40^ and a descriptive field study^41^ took place in supermarkets and targeted categories of 'less healthy' foods and drinks. The remaining study was a controlled field study^42^ which took place in a cafeteria and targeted SSDs. All studies were in adult populations and took place in the US. A controlled field experiment by Wansink^40^ randomly allocated households (*n* = 113) to either a control (no tax) or experiment (10% tax on 'less healthy' foods and drinks including all SSDs). The aim of the Wansink study was to assess the impact on SSD purchasing over a 6 month period. The results of the study showed a short term reduction in SSD purchase at 1 month, but this reduction was not seen at three or 6 months^40^. The results also indicated an increase in the purchases of alcohol. A similar type of descriptive field study by Elbel (purchases *n* = 3680)^41^, conducted in a store in a hospital, found that a 30% tax on unhealthy food resulted in an 11% higher chance of purchasing a ‘healthy’ food compared with baseline which was reflected in a significant reduction in the overall grams of sugar purchased^41^. The different locations and levels of taxations should be noted in comparing the results of these studies. The third study, by Block^42^, a controlled field study (*n* = 154) implemented a 35% tax on soft drinks (excluding diet drinks) in a hospital cafeteria. This resulted in a reduction of sales of regular soft drinks by 26% during the study period and increased to 36% during a combined tax and education period. Additionally there was an increase in sales of diet soft drinks by 20%. A 'control' site with no increase in price showed no change in soft drink sales during the same time period.

## Discussion

The aim of this rapid review was to examine the most recent evidence from experimental and observational studies from 2010 onwards on the health and behavioural impacts of fiscal measures targeted at high sugar food and SSDs. The vast majority of studies focused on impact in terms of consumer choice at the point-of-purchase. The evidence indicates that fiscal strategies may have an impact on sales/purchasing providing the tax levied is large enough.

The resulting evidence from 11 studies, of mainly moderate (*n* = 5) to high (*n* = 5) quality studies, suggests that increasing prices of high sugar foods and SSDs are likely to reduce purchases of these products, at least in the short term, and that this reduction may be somewhat proportionate to the level of price increase imposed. Data from almost all of the 11 experimental studies reviewed demonstrated that consumers can be responsive to changes in food and drink prices and where an effect was not reported, the 'tax' level was relatively low compared with the other studies. There was some consistency in the findings despite the diversity of approaches taken, which could suggest that the direction of the relationship is 'real' and not a result of low quality studies, unreliable statistics or small sample sizes. It is plausible that a reduction in purchases of high sugar foods and SSDs could result in a reduction in consumption and potentially drive population level reduction in sugar intake. However, no studies were found examining the effects of pricing on consumption or longer term health outcomes.

The lack of peer reviewed experimental evidence overall meant there was little robust evidence regarding effects that have been highlighted in the broader literature such as the potential difference in short vs. long term effects, the extent and nature of a regressive, and subsequently progressive, effect and an understanding of compensatory behaviours and their impact on individual and population level dietary intake and nutritional quality overall. There was limited evidence from one study by Darmon^33^ demonstrating a potential widening of inequalities in nutritional quality, as measured by energy density, quantity of free sugars and MAR, between medium-income and low-income groups as a result of a tax on unhealthy foods and a subsidy on healthy foods.

Several studies discussed the compensatory behaviours that resulted from their increased pricing of high sugar products and subsidising healthy products^33, 34, 38, 40, 42^. The compensatory behaviours reported depended on the target product, outcomes measured and the nature of the intervention. For example one study reported that reduced purchasing of SSDs also resulted in an increase in alcoholic drinks purchases^40^ and one study reported that reducing purchasing of unhealthy products resulted in reduced energy density overall but not significantly in relation to sugar^33^. The small number of studies, their heterogeneity and the variety of substitution effects observed suggests a need for caution in interpreting or attempting to generalise the findings.

Despite there being several countries where taxes on high sugar products are currently implemented, there were no evaluation studies from these countries that were eligible for inclusion in this review. Background reports in the grey literature highlighted sales and consumer panel data that have been used in several countries to suggest that there may be some short-term reduction in purchases resulting from current taxes, however, there are no data over extended time periods to show if these reductions are maintained. These data are however, supported by the results reported from experimental studies in this review. However, robust and transparent evaluations with a ‘natural experiment’ type of study design are needed before a causal effect between taxation and behaviour change for any of these countries can be determined^20^.

### This review in the context of the broader literature

The results from the literature review suggest that higher prices on targeted high sugar products do tend to reduce purchases of these products and that the size of the effect on purchasing levels may be proportionate to the size of the price rise implemented. This is supported by an extensive evidence base from modelled studies, not included in this review, that show that price changes are likely to influence purchasing^15, 22–25^. It is also supported, albeit non-conclusively, by the sales and consumer panel data that have been reported by some of the European countries that have recently implemented a tax^43, 44^.

Data analysed from the Euromonitor Passport Database by Ecorys^43^ found that demand for SSDs reduced by 4–10% as a result of fiscal strategies in Finland, France, and Hungary^44^. In addition, several modelling studies, not reviewed here, used sales data in US States where low level (<10%) taxes on SSDs or snack foods exist, to estimate effect on purchasing, consumption or health outcomes estimated small effect sizes. These studies and other econometric modelling studies have led a number of authors to estimate that a tax of between 10 and 20% is required to have an effect on behaviour and ultimately on population level health outcomes^10, 11, 26, 27^. This estimate is approximately supported by the result of this review which show that two studies with a smaller tax of <10% did not show an effect on purchasing. Higher taxes of <25% reported greater reductions in purchasing. However, it must be noted that the number of studies was small (*n* = 11) and heterogeneous. There were only four (out of 11) studies that tested the effect of a <25% price increase and only one of these targeted a solely high sugar product. Prior to the current review only one systematic review, by Epstein^28^ of experimental studies examining the impact of fiscal studies had been published, this had a broader inclusion criteria which included foods other than sugar and was over an earlier and wider date range (1980–2011). However, the findings from the Epstein^28^ review align with the findings presented here, concluding that price changes can modify purchases of targeted foods. Although the impact on overall dietary intake and quality, including any substitution effects, remains poorly reported and requires further research.

### Limitations of this review

The included evidence was a small number (*n* = 11) of short term, heterogeneous, international studies of variable quality that measured impact in relation to purchasing outcomes. The international aspect of the included studies should be noted and extrapolating findings to individual countries, such as the UK, should be done with caution. Few studies gave adequate information about randomisation and blinding methods. Virtual studies may not adequately reflect a real life setting. The complex, multi-dimensional nature of the subject matter being explored does not necessarily lend itself well to laboratory-style studies or randomised controlled trials in localised settings such as hospital cafeterias. More robust evidence from empirical data is needed to ensure that there is not an over reliance on modelling and simulation studies^45^, but careful planning and consideration is required to ensure that causality between fiscal measure based interventions, behaviour change and health outcomes can be demonstrated^8^. More pragmatic approaches should be taken to evaluating the effectiveness of taxing high sugar products whilst ensuring that causality, substitution effects, impact on lower income groups and sustainability can be robustly assessed. There was a paucity of studies that examined the effect of price increases on children and adolescents or the impact on different socio-economic groups.

It is important to consider the findings presented in this review within the following methodological limitations:

1. This review specifically focused on evidence from high sugar foods and SSDs; however, much of the research evidence is focused on broader groups such as unhealthy, energy dense, high calorie for nutrients, high fat, sugar and salt products and these studies will not have been identified for inclusion unless they provided a specific reference to a high sugar component. This may have limited the size and range of the evidence base assessed.
2. Given the requirement to identify and examine a range of interventions and outcomes, in both adult and child populations, a broader more flexible approach had to be applied to the review methodology (see full research brief in Appendix 1).
3. Due to time and resource restraints, only one reviewer conducted the initial reference screening. Gold standard systematic review protocols such as Cochrane and JBI recommend second reviewer screening to help reduce the likelihood of missing relevant studies and introducing selection bias.
4. Restricting studies by date (2010 onwards), English language, and to experimental and observational design only will have limited the range of the evidence base reviewed. The language restriction could have limited possible learning from non-English speaking countries.

### Research recommendations

The evidence presented in this report highlights a number of areas for future research consideration, namely more high quality research carried out in both adult and child populations with a focus on (1) The short, medium and long term impacts (including evaluation of patterns of impact variability over time) of fiscal measures relating to high sugar foods and SSDs on behavioural and health outcomes in free-living individuals. Currently studies examine sales or purchasing behaviour. Ultimately, it is the impact of manipulating taxes on consumption behaviour and health outcomes that is of interest and needs to be studied; (2) The impact on population sub groups and resulting impact on inequalities particularly with regards to the potentially regressive/progressive nature of a tax on high sugar food and non-alcoholic drink; (3) The impact of compensatory and substitution effects and other associated behaviours; (4) Attitudes towards the implementation and acceptability of a tax.

## Conclusion

Findings from this review suggests that increasing prices of high sugar foods and SSDs, potentially through taxation, is likely to reduce purchases of these products in the short term. The empirical data assessed in the included studies reviewed demonstrated that consumers are responsive to changes in food and drink prices and where an effect was not reported, the tax was relatively low compared with other studies. These findings complement the evidence from modelling studies which indicate that taxation would lead to a reduction in purchases proportionate to the level of tax applied. Moreover, the available evidence on sales data from countries that have implemented a tax on sugar products also aligns with these findings to suggest that purchases have reduced since the tax was implemented. The current evidence base appears to converge and suggests that a fiscal strategy is likely to reduce purchases of high sugar products at least in the short term. However, the overall lack of peer-reviewed experimental and observational evidence has resulted in very little insight into associated effects that have been highlighted in the broader literature such as the difference in short and long term effects, the extent and nature of a regressive (and progressive) effect and an understanding of compensatory behaviours and their impact on individual and population level dietary intake and nutritional quality overall. Any new tax should be accompanied by a robust evaluation which examines the long term effects of any price increases, specifically assessing compensatory behaviours and whether price increases would exacerbate health inequalities within certain population subgroups.

## Electronic supplementary material

Appendices
