# Supplementary material for: A rapid review examining purchasing changes resulting from fiscal measures targeted at high sugar foods and sugar-sweetened drinks
Source: Nutr Diabetes. 2017 Dec 15;7(12):302. doi: 10.1038/s41387-017-0001-1 (PMC5865540; doi:10.1038/s41387-017-0001-1)
Supplement: Supplementary file 1 — Appendices [file 41387_2017_1_MOESM1_ESM.docx]

**APPENDICES:**

**APPENDIX 1: Research brief**

PHE carried out this review in collaboration with Teesside University to bring together the most recent robust evidence in this area to allow in-depth consideration of a possible policy initiative to reduce sugar consumption. It is intended to contribute to the package of evidence to inform the government’s thinking on sugar in the diet as requested by the Department of Health. A broader and rather more flexible approach was therefore needed that still adhered to a systematic methodology, but did not strictly follow the conventional Joanna Briggs Institute (JBI) or Cochrane approaches to systematic reviews.

Pragmatic decisions were therefore made regarding the methodology and inclusion criteria by a project steering group to ensure it fulfilled these requirements. These decisions included: developing evolving inclusion criteria, which were looser than would be expected of an academic publication to ensure that the outcomes supported policy thinking; literature searches were limited to 2010 onwards to ensure studies were most relevant to present day environment; interviews with key informants were included to support the literature review, as it was thought much evidence in relation to evaluation of existing fiscal measures implemented internationally may not yet have been published. Modelling and qualitative studies were excluded as modelling studies have been reviewed elsewhere and a decision was made to focus on only the most robust empirical quantitative studies. The findings of this review are, however, placed within the context of the wider literature in the general discussion. It was not within the scope of this research to consider the legal implications of implementing fiscal measures.

**APPENDIX 2: EXAMPLE SEARCH STRING**

Database: Ovid MEDLINE(R) In-Process & Other Non-Indexed Citations, Ovid MEDLINE(R) Daily, Ovid MEDLINE(R) and Ovid OLDMEDLINE(R) <1946 to Present>

Search Strategy:

--------------------------------------------------------------------------------

1 sugar$.mp. [mp=title, abstract, original title, name of substance word, subject heading word, keyword heading word, protocol supplementary concept word, rare disease supplementary concept word, unique identifier]

2 (sugar adj sweetened).mp.

3 (added adj sugar$).mp.

4 (sugar adj3 contain$).mp.

5 exp "food and beverages"/

6 drink.mp.

7 drinks.mp.

8 (soft adj drink$).mp.

9 fizzy.mp.

10 cake$.mp.

11 pastr$.mp.

12 biscuit$.mp.

13 pudding$.mp.

14 dessert$.mp.

15 preserve$.mp.

16 jam$.mp.

17 marmalade$.mp.

18 confectionary.mp.

19 chocolate$.mp.

20 sweet$.mp.

21 exp Energy Drinks/

22 (sport$ adj drink$).mp.

23 yog?urt$.mp.

24 (breakfast adj cereal$).mp.

25 juice.mp.

26 cordial$.mp.

27 squash$.mp.

28 snack$.mp.

29 candy.mp.

30 soda.mp.

31 bake$.mp.

32 1 or 2 or 3 or 4 or 5 or 6 or 7 or 8 or 9 or 10 or 11 or 12 or 13 or 14 or 15 or 16 or 17 or 18 or 19 or 20 or 21 or 22 or 23 or 24 or 25 or 26 or 27 or 28 or 29 or 30 or 31

33 fiscal.mp.

34 exp Taxes/

35 tax.mp.

36 taxes.mp.

37 taxing.mp.

38 exp Jurisprudence/

39 law.mp.

40 laws.mp.

41 price$.mp.

42 pricing.mp.

43 subsid$.mp.

44 exp Legislation, Food/

45 legislat$.mp.

46 exp Policy/

47 Policy Making/

48 33 or 34 or 35 or 36 or 37 or 38 or 39 or 40 or 41 or 42 or 43 or 44 or 45 or 46 or 47

49 obes$.mp.

50 over?weight.mp.

51 (weight adj3 gain).mp.

52 adipos*.mp.

53 (tooth adj decay*).mp.

54 (dental adj (caries or cary or carie)).mp.

55 exp Oral Health/

56 nutrition.mp.

57 exp Food Preferences/

58 exp Diet/

59 exp Energy Intake/

60 caloric.mp.

61 calorific.mp.

62 calorie.mp.

63 impact.mp.

64 49 or 50 or 51 or 52 or 53 or 54 or 55 or 56 or 57 or 58 or 59 or 60 or 61 or 62 or 63

65 32 and 48 and 64

66 limit 65 to (english language and yr="2004 -Current")

**APPENDIX 3. Quality assurance summary tables.**

Colour coding: Green: yes, Red: no, Yellow: unclear; Clear (no fill) N/A

| RCTs and pseudo-randomised trials assessments | | | | | | | | | |
| --- | --- | --- | --- | --- | --- | --- | --- | --- | --- |
| Study | Was the assignment to the treatment group truly random? | Were participants blinded to treatment allocation? | Was allocation to treatment groups concealed from the allocator? | Were the outcomes of people who withdrew described and inluded in the analysis? | Were those assessing outcomes blind to the treatment allocation? | Were the control and treatment groups comparable at entry? | Were groups treated identically other than for the named interventions? | Were outcomes measured in a reliable way? | Was appropriate statistical analysis used? |
| Waterlander^(39)^ |  |  |  |  |  |  |  |  |  |
| Waterlander^(40)^ |  |  |  |  |  |  |  |  |  |
| Giesen^(33)^ |  |  |  |  |  |  |  |  |  |
| Nederkoorn^(41)^ |  |  |  |  |  |  |  |  |  |

| Comparable cohort studies: | | | | | | | | | |
| --- | --- | --- | --- | --- | --- | --- | --- | --- | --- |
| Study | Is sample representative of participants in the population as a whole? | Are the participants at a similar point in the course of their condition? | Has bias been minimised in relation to selection of cases and of controls? | Are confounding factors identified and strategies to deal with them stated? | Are outcomes assessed using objective criteria? | Was follow up carried out over a sufficient time period? | Were the outomes of people who withdrew described and inluded in the analysis? | Were outcomes measured in a reliable way? | Was appropriate statistical analysis used? |
| Wansink^(42)^ |  |  |  |  |  |  |  |  |  |
| Block^(32)^ |  |  |  |  |  |  |  |  |  |

| Descriptive studies: | | | | | | | | | |
| --- | --- | --- | --- | --- | --- | --- | --- | --- | --- |
| Study | Was study based on a random or pseudo-ramdom sample? | Were the criteria for inclusion in the sample clearly defined? | Were confounding factors identified and strategies to deal with them stated? | Were outcomes assessed using objective criteria? | If comparisons are being made, was there sufficient descriptons of the groups? | Was follow up carried out over a sufficient time period? | Were the outcomes of people who withdreew described and included in the analysis? | Were outcomes measured in a reliable way? | Was appropriate statistical analysis used? |
| Darmon^(36)^ |  |  |  |  |  |  |  |  |  |
| Temple^(34)^ |  |  |  |  |  |  |  |  |  |
| Epstein^(37)^ |  |  |  |  |  |  |  |  |  |
| Salvy^(38)^ |  |  |  |  |  |  |  |  |  |
| Elbel^(35)^ |  |  |  |  |  |  |  |  |  |

**APPENDIX 4. Data summary tables**

**Key:**

COLOUR CODES: blue shading: adult studies; pink shading: child studies.

IMPACT CODES: +: intervention had a positive impact on reducing sugar; - intervention had a negative impact on reducing sugar (i.e. sugar increased); 0 intervention had no impact on reducing sugar.

* This refers to the number of quality assurance (QA) criteria met using the Joanna Briggs Institute criteria (each score is out of 9 but if a criteria is marked as not applicable it is deducted from the total)^(31)^

** population details are provided only when available.

f=female

m=male

SES=socio-economic-status

SSDs/SSBs=Sugar-sweetened drinks/Sugar-sweetened beverages

VAT=Value Added Tax

*Italics: denote studies that report high sugar foods as part of the list of target foods/products but do not present separate outcomes for the high sugar foods alone. This reflects the breadth and variety of outcomes in the literature and the fact that outcomes measures were often pragmatically defined to reflect how sugar in purchased and consumed as part of a meal in the overall diet of free living individuals. The authors acknowledge that these studies were identified from a literature search focused on ‘high sugar’ impact but where it was only apparent on full review that the impact of the high sugar food is not reported in isolation, but as part of the impact analyses for all the products or meal. It is therefore possible that other studies not included in this review may include impact data on more broadly defined categories of foods that are for example high in fat, sugar and salt, but were not selected as the title and abstract did not suggest or refer to the ‘high sugar’ component specifically.*

| **Author (date), country** | **Study type (size) & population**** | **Outcomes measured: [O: objective; S: subjective]** | **Intervention aim, method & target product** | **Authors’ Key finding** | **QA score***  **[impact: +/-/0]** |
| --- | --- | --- | --- | --- | --- |
| **LABORATORY/VIRTUAL EXPERIMENTS** | | | | | |
| Darmon (2014), France  ^(36)^ | Experimental study, laboratory virtual shopping basket.  F, low income (n = 95), mean age 35.3 ±7.0; medium income (n=33), mean age 34.8 ±6.9 | i) Total quantities of food (beverages included) for each class (fruit and vegetables, other healthy foods, neutral products and unhealthy  products)  ii) Quantities of beverages only  iii) Total expenditures (based on posted prices)  iv) Dietary quality indices  [Energy density (kcal/100g), free sugars (% energy) and the mean adequacy ratio (MAR) were used as nutritional quality indicators.]  [O] | The study aim was to assess the selections made by the sample in a daily food basket, first, at current prices then at manipulated prices.  Two price manipulations were examined:   1. a fruit and vegetable price subsidy named “fruit and vegetables condition” 2. a 30% healthy-product subsidy coupled with a 30% unhealthy-product tax named “nutrient profile condition”.   Target foods/products:  ‘healthy’/’unhealthy’ foods | The results from the ‘nutrient profile condition’ showed that subsidising healthy products and taxing unhealthy products improved some aspects of the nutritional quality of food choices in both income groups. It especially reduced the energy density of the individual daily food baskets selected by low-income and medium-income women and in doing so tended to reduce the total energy content of the baskets.  Low-income women derived fewer nutritional benefits from the condition than medium-income women. Authors conclude that this outcome suggests that food price policies may improve diet quality while increasing socio-economic inequalities in nutrition.  [Outcome data for added sugars presented in paper show that less added sugar was consumed after each manipulation in both income groups but these differences were not significant].  Statistical tests  Regression was used to compare the means of each variable after adjustment for the total energy content of selected daily basket. All analyses were stratified by income groups. | 4/9  [+] |
| Waterlander (2014), Netherlands  ^(39)^ | RCT, 3-D Virtual supermarket.  n=102 (95 results analysed, 49 control, 26 experimental, f=77%, 59% students, mean age 28.7 years ± 13.8). | Purchased quantity (litres) of SSDs per household per week.  [O] | The aim of this study was to examine the effects of a price increase (to reflect an increase in Dutch VAT from 6%-19%) on SSDs on beverage and snack purchases  2x conditions:   1. Experimental condition 19% tax on SSDs (increase of Dutch VAT from 6% to 19%) 2. Control condition regular prices (Dutch VAT at 6%)   Target food/product: SSDs | This study showed that a higher VAT on SSD resulted in statistically significantly lower SSD purchases in an acute experimental setting.  A VAT increase on SSD from 6% to 19% was effective in decreasing SSD purchases and had no significant effects on other beverage categories (including alcohol) or snack foods.  Statistical tests   1. Crude differences between experimental and control conditions in purchased quantities analysed using independent t-tests. 2. Examined whether sex, price perception (high/low), budget (high/low), student (yes/no), and level of habit strength (high/low) modified the associated between the intervention and SSD purchase. 3. Two regression models were constructed for all outcome measures; one accounting for basic confounders sex, student, and shopping budget, and one accounting for realism/feasibility score for the virtual supermarket, level of habit strength, and price perception score. | 8/9  [+] |
| *Waterlander (2012). Netherlands*  *^(40)^* | *RCT, web based supermarket.*  *n=117, f, aged 18+, low SES.* | *Primary:*  *Purchases of ‘healthy’ and ‘unhealthy’ foods (items and %), fruit and vegetables (gram).*  *Secondary:*  *Purchases of healthy products fruit and vegetables (number and %), proportion.*  *[O]* | *The study aim was to examine the effectiveness of varying taxing and subsidising schemes to stimulate healthier food purchases.*  *Web based supermarket with 3 levels of price reduction for ‘healthy’ foods (no; 25%; 50%) x 3 levels of price increase on ‘unhealthy’ foods (5%; 10%; 25%) factorial design was used.*  *Target foods/products:*  *‘Healthy’ and ‘unhealthy’ products defined by WHO nutritional labelling guidelines regarding sat fat, trans fat, sodium, added sugar).* | *Participants receiving 50% discount purchased significantly more ‘healthy’ foods then subjects receiving no discount (mean difference = 6.62 items, p<0.01) or 25% discount (mean difference = 4.87 items, p<0.05).*  *Participants in the highest discount also purchased significantly more calories.*  *Price increases ‘unhealthy’ products did not significantly affect food purchases.*  *Statistical tests*  *Mean differences in outcome measures between conditions were tested using a two-way factorial ANCOVA, where the factor 1 indicated the level of discount and factor 2 the level of price increase.* | 8/9  [0] |
| *Giesen (2011), US.*  *^(33)^* | *Pseudo-RCT (within-participant groups <20, randomly assigned to 4 different conditions).*  *Opportunistic sample of students, n=178 students (95 men).* | *Baseline: ‘Hunger rating’, ‘Restraint Scale’, BMI*  [O] | *The aim of the study was to test the combined effects of providing calorie information and increased taxes on the total number of calories purchased for lunch. These conditions were tested in the context of high and low ‘restrained eaters’.*  *3 x separate selections from hypothetical lunch menu. High-calories foods prices were increased each time:*  *1st as per university menu*  *2^nd^ 125%*  *3^rd^ 150%*  *In addition 3 x between subject conditions:*   - *Budget (high/low)* - *Calorie information (information/no information)* - *Dietary restraint*   *Target foods/products:*  *‘High calorie’ foods (categorised into more and less expensive)* | *Regression analysis showed a significant main effect from tax in the ‘no calorie labelling’ condition (estimate = -0.078, p<0.001) which indicated that a price increase for high-calorie food products was associated with a decrease in calories purchases.*  *“A food tax of >= 25% makes nearly everyone buy fewer calories.”*  *(No outcome data specific to sugar.*  *Brownies, glazed donuts, chocolate candy bar and SSDs described in menu list).*  *Statistical tests*  *Mixed effects regression models were used to estimate the elasticity of calories as a function of price.* | *6/9*  *[+]* |
| *Nederkoorn (2011), Netherlands*  *^(41)^* | *Experimental, between-subject RCT, n=306, mean age 41.2.*  *Virtual web-based supermarket.* | *Baseline: Daily budget on food,*  *Momentary hunger*  *Outcomes: Food purchasing behaviour (using web based supermarket task)*  *[O]* | *The aim of the study was to examine whether a high tax on high calorie dense foods effectively reduces the purchased calories of high energy dense foods in a web based supermarket and whether this effect is moderated by budget and weight status.*  *Participants randomly assigned to either control (normal prices) or experimental (50% tax condition on ‘High Energy Density’ foods).*  *Target foods/products:*  *‘High Energy Density’ foods (all products with a caloric value of more than 300 kcal/100g).*  *.* | *Results showed that relative to the no tax condition, the participants in the tax condition bought less calories. The main reduction was found in high energy dense products and in calories from carbohydrates, but not in calories from fat. A 50% tax on High Energy Density (HED) foods caused a 16% decrease of purchase in these products. BMI and budget did not influence the effectiveness of the tax. The reduction in calories occurred regardless of budget or BMI implying that a food tax may be a beneficial tool, along with other measures, in promoting a diet with few calories. The HED tax predicted the purchase of calories significantly. Specifically, the tax diminished the purchase of total calories (ΔR^2^ =.021, p<.01).*  *(No outcome data specific to sugar but ‘High Energy Density’ taxed foods list included chocolate, cookies and sweets).*  *Statistical tests*  *Effect of tax was analysed by 7 hierarchical regression models with:*  *Total calories, calories from high energy dense products, calories from low energy dense products, percentage calories from high energy dense products and calories from carbohydrates, fat, protein as dependent variables.* | 6/9  [+] |
| *Temple, (2011), New York.*  *^(34)^* | *2 experimental studies, within-subject design,*  *Age 18-50 years, opportunistic sample recruited from University students, staff, community members using flyers.*  *Experiment 1: n=35 overweight or obese, n=16 lean.*  *Experiment 2: n=20 obese, n=21 non-obese.* | *Baseline: Demographics, anthropometrics, basic medical info, food preferences*  *Outcomes: Nutrition information condition of food purchased (No label, standard label, ‘Traffic Light Diet’ label), Energy intake.*  *[O]* | *The aim of the study is to test hypotheses that simplified nutrition labelling and taxation (independently) alter food selection and intake.*  *Participants received a brief education session on the labelling system being used.*  *Experiment 1: participants consumed lunch on 3 occasions in lab under 3 x conditions:*  *1. No nutrition labels*  *2. Standard nutrition labels*  *3. ‘Traffic Light Diet’ labels.*  *Experiment 2: participants were randomised to a ‘no label’ or a ‘Traffic Light Diet’ label condition. They were given $6 to purchase lunch in lab under 2 x conditions:*  *- all foods with standard pricing*  *- 25% tax on ‘red’ foods*  *Target foods/products:*  *‘Traffic Light Diet’ labelled foods:*  *‘Green’ = foods low in calories, low added sugar <10% calories/serving; ‘Yellow’ = moderate calories, added sugar 10-25% calories/serving;*  *‘Red’ = high calories, added sugar >25% calories/serving, fat>5g/serving.* | *Results for experiment 2 (the ‘tax’ experiment) showed that for ‘red’ food purchases there was a main effect of taxation condition on number of ‘red’ items purchased (F(1,39) = 6.9; p=0.012), with taxation reducing the purchasing of ‘red’ foods. There was also an interaction between weight status and taxation on purchasing of ‘red’ foods (F (1, 39) = 4.1; p=0.049), with taxation reducing red food purchases in obese participants, but not in non-obese participants.*  *(No outcome data specific to sugar purchases were reported however the following foods were listed under the ‘red’ food label: gelatine dessert, granola bars, chocolate sandwich cookies, candy bars, chocolate milk, lemonade, sports drinks, regular sodas).*  *Statistical tests (experiment 2)*  *Number of food items purchased from ‘red’, ‘yellow’ and ‘green’ food categories were analysed using a mixed analysis of covariance: between-subject factors: label condition (no label or ‘Traffic Light Diet’ label), weight condition (obese or non obese), order of taxation condition and gender; within-subjects factor: taxation condition; covariates: Binge Eating Scale and age.* | 7/8  [+] |
| *Epstein (2010), US*  *^(37)^* | *Experimental observational study (within-subject design), analogue supermarket purchasing study. .*  *n=42, f, (n=20 low income), recruited via university database, screened for a) responsibility for grocery shopping for family b) at least one child 6-18yrs old, 45% obese, 23.8% ‘minority status’.* | *Purchases of foods/products*  *[O]* | *The aim of the study was to examine effects of increasing the price of ‘less healthy’/‘high-calorie-for nutrient’ (HCFN)*  *foods or reducing the price of ‘more healthy’/low-calorie-for-nutrient (LCFN) foods by 12.5% and 25% respectively on 68 common foods.*  *Participants were given $22.50 and told to purchase food for the family when there was nothing at home. Each participants completed 5 purchasing tasks:*   1. *Prices were based on current grocery store prices* 2. *LCFN foods lowered by 25%* 3. *LCFN foods lowered by 12.5%* 4. *HCFN foods 25% (LCFN unchanged)* 5. *HCFN foods 12.5% (LCFN unchanged)*   *Target foods/products:*  *68 common foods categorised into HCFN/LCFN based on the ‘Calories For Nutrients’ index (calculates calories required to gain 1% of recommended daily value of 13 key nutrients)* | *Taxing HCFN foods resulted in a significant decrease in total energy purchased (elasticity estimate = -0.65, p<0.0001).*  *Taxing foods reduced purchases of HCFN foods while increasing purchases of LCFN foods.*  *Taxing of HCFN foods resulted in a significant reduction in fat and carbohydrates and the proportion of calories from fat (elasticity estimate=-0.12, p=0.005) and an increase in the proportion of calories from protein (elasticity estimate=0.098, p<0.0001). The improvements of the quality of the diet when HCFN foods were taxed were associated with significant reductions in purchases of HCFN foods (elasticity estimate=-1.44, p<0.0001).*    *10% increase in price of HCFN foods resulted in 6.5% less calories purchased and 14.4% less HCFN foods purchased.*  *(No outcome data specific to purchases of sugar however high sugar products are listed as included in HCFN ‘taxed’ foods such as candy, cookies, ice cream and SSDs)*  *Statistical tests*  *Effects of the ‘tax’/’subsidy’ conditions on purchases of LCFN/LCFN foods were analysed using mixed effects regression models.* | 4/7  [+] |
| *Salvy (2012), US*  *^(38)^* | *Experimental observational study, analogue convenience store purchasing task.*  *All aged 12-14 years, BMI above 5^th^ percentile and below 95^th^ percentile.*  *Experiment 1: n=37, 18 m, 19 f.*  *Experiment 2: n=52, 26 m, 26 f.* | *Purchases of foods/products.*  *[O]* | *The aim of the study is to examine the impact of taxes (and subsidies) on purchases of healthy and unhealthy snack food in adolescents.*  *7 HCFN and 7 LCFN snacks were displayed in experimental room. Each participant completed 9 x purchasing tasks; 1 x prices as per local convenience stores, 4 x HCFN prices were fixed while prices of LCFN were raised (‘taxed’) or lowered (‘subsidised’) by 25% or 50%, 4 x LCFN snack prices were fixed and prices of HCFN snacks were raised or lowered by 25/50%. Participants were given a study income of $3 and were told to imagine they were hungry as they were coming home from school and had stopped at a store for something to eat.*  *Experiment 1: examined behaviour when participant was alone.*  *Experiment 2: examined behaviour when participant was in the presence of a same age, same sex peer.*  *Target foods: ‘High-calorie-for-nutrient’ (HCFN) snack foods, including crisps, cookies, chocolate bars and sweets.*  *(Calories-for-nutrients was assessed with the CFN index.)* | *Price manipulations affect adolescents’ spending on snack foods.*  *Taxing unhealthy food results in less unhealthy food purchased in both experiments.*  *Experiment 1: fewer kilocalories of HCFN snacks purchased were predicted by taxing HCFN snacks (β=-5.13, SE=1.08, p<0.001).*  *Experiment 2: fewer kilocalories of HCFN snacks purchased were predicted by taxing HCFN snacks (β=-4.32, SE=0.97, p<0.001).*  *(No outcome data specific to purchases of sugar (grams) however many high sugar products are listed as included in HCFN ‘taxed’ foods such as cookies, sweets and chocolate bars)*  *Statistical tests*  *Univariate and multivariate mixed regression analyses examined the relationship between snack price and the amount of HCFN and LCFN snack purchases.* | 4/9  [+] |

| **SUPERMARKET EXPERIMENTS** | | | | | |
| --- | --- | --- | --- | --- | --- |
| **Author (date), country** | **Study type (size) & population**** | **Outcomes measured: [O: objective; S: subjective]** | **Intervention & target product** | **Authors Key finding** | **QA score***  **[impact: +/-/0]** |
| Elbel (2013), US.  ^(35)^ | Controlled field experiment (no tax = control).  Study size based on number of purchases (n=3680) | Purchases of ‘less healthy’ foods/products.  [O] | The aim of the study is to determine whether ‘taxing’ (or labelling) of ‘unhealthy’ food influenced purchasing in a controlled field experiment.  Purpose built experimental corner store open to public in out-patients areas of a large hospital in New York. 3680 purchases made during the experiment.  5 conditions:   1. Baseline no labelling 2. 30% tax on ‘less healthy’ foods 3. Labelled ‘less healthy’ 4. Tax and ‘less healthy’ label 5. Tax and label stating higher price due to ‘less healthy’   Target foods/products:  ‘Less healthy’ or ’healthy’ foods. ‘Less healthy’ foods were categorised (amongst other criteria) as ≥35% total weight from sugar). | Consumers were 11% more likely to purchase a ‘healthier’ item under a 30% tax on ‘less healthy’ (95% CI 7%, 16%, p<0.001).  By product type, consumers switched away from the purchase of ‘less healthy’ foods under taxation (9 percentage point decrease, p<0.001) and into healthier beverages (6 percentage point increase, p0.001); there were no effects for labelling.  Any of the tax conditions on ‘less healthy’ foods, with or without labelling, were associated with the purchase of 2 fewer grams of sugar (p<0.001). Results remained significant controlling for all items purchased in a single transaction.  Statistical tests  Logistic regression used to analyse the association between various conditions and purchases of a ‘healthier’ item at both the single-item and transaction levels. | 7/7  [+] |
| Wansink et al (2012), USA  ^(42)^ | Randomised controlled field experiment.  113 households randomly assigned to either a 10% tax on SSBs or control group.  Final sample = 89 households (total number of purchase observations = 623) | Observation of purchases of SSBs over 7 months.  [O] | The aim of the study was to examine how ‘taxing’ of SSBs would impact on consumer purchasing (and thus potentially consumption) behaviour.  The randomly assigned ‘taxation’ group experienced a 10% tax on all ‘less healthy’ (defined as ‘few nutrients per calories) were taxed (including all SSBs).  Alcohol was not discounted or taxed. | The tax resulted in a short-term (1 month) decrease in SSB purchase. There was no decrease over the 3 or 6 month period. There was an increase in beer purchases in the tax group.  Concluded that a 10% tax on less healthy foods did not produce a significant change in SSB purchase at 3 or 6 months and there is evidence of unintended consequences in the form of results showing an increase in alcohol purchases.  Authors recognise that as all ‘less healthy’ foods were taxed, there were other potential substitution effects that were not examined.  Statistical tests  Random effects panel regression technique with robust standard errors was used to analyse the data to estimate the impact of the tax on the volume of SSB purchases. | 7/8  [0] |
| **CAFETERIA/RESTAURANT EXPERIMENTS** | | | | | |
| Block (2010), US  ^(32)^ | Controlled field experiment  (Within-subject design).  n=154 | Primary outcomes: daily sales of soft drinks, diet soft drinks, zero calorie water.  Secondary outcomes: sales of other categories of beverages.  [O] | The aim of the study is to investigate whether a price increase on regular (sugary) soft drinks and an educational intervention would impact on sales.  5-phase intervention in a hospital cafeteria:  1) Baseline  2) 35% tax on regular soft drinks 3) reversion to baseline prices (washout)  4) educational campaign  5) combination tax and education.  Target foods/products: Data collected from a comparison site, for the final 3 phases*.* | Sales of regular soft drinks decreased by 26% during the price increase phase (95% CI = 39.0, 14.0). This reduction in sales persisted throughout the study period and was increased to 36% during the combination phase (95% CI=49, 23).  Diet soft drinks sales increased during this period by 20% (95% CI=7.0, 33.0) Education had no independent effect on sales. Analysis of the control comparison site showed no change in regular soft drink sales during the study period.  Statistical tests  Regression models with each of the primary and secondary outcomes, expressed as daily sales, as dependent variables. | 4/8  [+] |
